# Data-driven probabilistic mapping of the spatial and molecular landscape of glioma

**DOI:** 10.1093/braincomms/fcaf459

**Published:** 2025-11-26

**Authors:** Nardin Samuel, Jurgen Germann, Andrew Yang, Can Sarica, Alexandre Boutet, Artur Vetkas, Brendan Santyr, Stefan Lang, Davide Chicco, Andres M Lozano

**Affiliations:** Division of Neurology, Department of Medicine, University of Toronto, Toronto, ON M5S 1A8, Canada; Krembil Brain Institute, University Health Network, Toronto, ON M5G 2C4, Canada; Krembil Brain Institute, University Health Network, Toronto, ON M5G 2C4, Canada; Institute of Biomedical Engineering, University of Toronto, Toronto, ON M5S 3G9, Canada; Krembil Brain Institute, University Health Network, Toronto, ON M5G 2C4, Canada; Krembil Brain Institute, University Health Network, Toronto, ON M5G 2C4, Canada; Department of Radiology, University of Toronto, Toronto, ON M5G 2C4, Canada; Department of Neurosurgery, Tartu University Hospital, Tartu 50406, Estonia; Division of Neurosurgery, Toronto Western Hospital, Toronto, ON M5T 2S8, Canada; Division of Neurosurgery, Department of Surgery, University of British Columbia, Vancouver, BC V5Z 1M9, Canada; Dipartimento di Informatica Sistemistica e Comunicazione, Università di Milano-Bicocca, Milan 20126, Italy; Institute of Health Policy Management and Evaluation, University of Toronto, Toronto, ON M5T 3M6, Canada; Krembil Brain Institute, University Health Network, Toronto, ON M5G 2C4, Canada; Division of Neurosurgery, Department of Surgery, University of Toronto, Toronto, ON M5S 1A8, Canada

**Keywords:** glioma, radiology, probabilistic mapping

## Abstract

Understanding the spatial distribution of gliomas in the brain and their molecular subtypes can aid in the diagnosis and development of targeted therapies. This study aims to create probabilistic radiologic maps of glioma locations using large MRI datasets and the most recent consensus brain tumour classification. Neuroimaging data from multiple databases were analysed. Patients included had MRI T1 images and validated tumour segmentations. Probabilistic tumour maps were generated whereby binary tumour masks were aligned to a standard brain template and aggregated to compute voxel-wise frequency maps of glioma occurrence detailing glioma volume, molecular subtype, age, sex and overall survival with tumour location. The study included 2164 patients with gliomas. Key findings include distinct spatial patterns associated with glioma size and molecular subtype: smaller tumours favoured the left temporal region, medium-sized tumours the medial frontoparietal and bilateral temporal regions and larger tumours the frontotemporoparietal regions, predominantly on the right. Isocitrate dehydrogenase (IDH)-wild-type tumours were more common in medial parietotemporal regions, while IDH-mutant tumours were preferentially found in frontotemporal regions. Younger patients had more frontal tumours, while older patients had higher parieto-occipital tumour burdens. Tumours in medial structures and parietal lobes were linked to lower survival, whereas right temporal tumours were associated with higher rates of survival. These findings likely correlate with IDH mutation status. Leveraging eight glioma databases, probabilistic tumour maps revealed significant relationships between brain regions, molecular subtypes and clinical outcomes. These findings could be used in clinical decision-making and offer insights into glioma pathogenesis and treatment of patients impacted by this disease.

## Teaser

Samuel *et al*. report probabilistic brain maps of glioma derived from over 2000 patients across eight datasets. Tumour location was linked with molecular subtype, age and survival. These findings provide a large-scale resource to improve diagnosis, prognostication and understanding of glioma pathogenesis and treatment strategies.

## Graphical Abstract

**Graphical Abstract:**
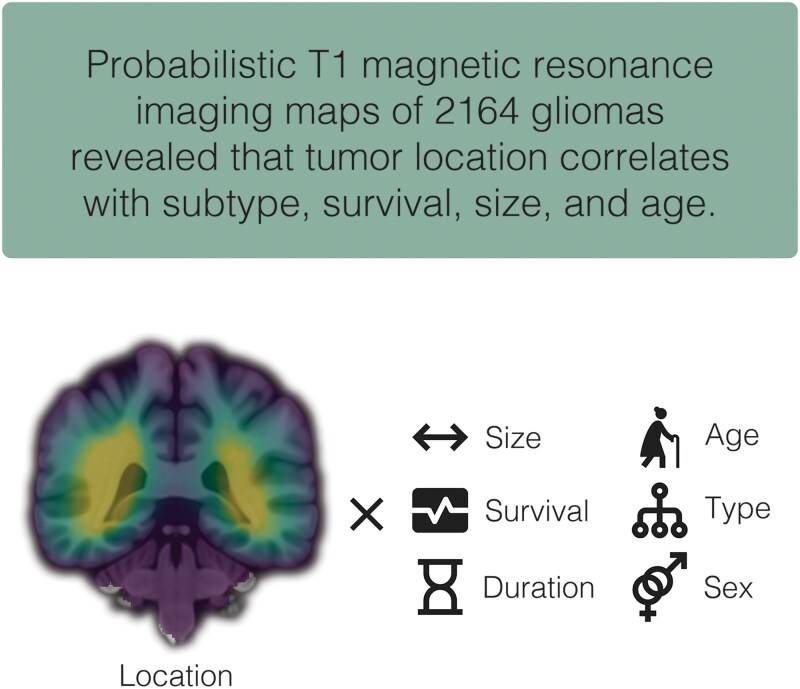

## Introduction

Gliomas are the most common primary brain tumours in adults, with an annual incidence of 5–10 per 100 000 individuals, and despite advances in treatment, the prognosis for patients with high-grade gliomas remains poor, with a median overall survival of 12–15 months.^1^ Although radiographic imaging is central to their clinical management, the spatial distribution of gliomas and its relationship with neuroanatomical structures are poorly understood. Given the infiltrative properties of gliomas, emerging evidence suggests that these tumours follow intrinsic structural and functional networks during their progression. Previous studies have suggested that gliomas may preferentially arise in specific regions of the brain, such as the periventricular zone and the insula.^2-6^ Moreover, accumulating evidence indicates that histological and molecular subtypes of glioma may also have distinct spatial distributions.^7-10^

Identifying regions with a propensity for harbouring gliomas may shed light on the underlying factors that influence tumour initiation and progression, such as the unique microenvironmental features and connectivity profiles of different brain areas.^11^ Importantly, the spatial distribution of gliomas in relation to brain networks can also inform the interplay between glioma biology and brain network architecture. As such, mapping the location and patterns of spread of glioma within the brain is crucial for understanding their pathophysiology and developing effective treatment strategies. By leveraging network mapping approaches, the anatomical distribution and heterogeneity of these tumours can be characterized at a systems level. Moreover, integrating advanced neuroimaging techniques with molecular profiling of tumours holds potential for elucidating the complex interactions between gliomas and the surrounding neural circuitry.

Towards this, the present study aimed to derive probabilistic radiologic maps of glioma locations using large datasets of MRI scans from patients with newly diagnosed gliomas. This approach enabled the identification of regions of the brain with a higher likelihood of harbouring gliomas. To our knowledge, this is one of the largest multi-dataset studies to date using this methodology, providing a novel data-driven perspective on glioma spatial patterns. Further, in contrast to other studies, this study employed the most recent WHO brain tumour classification system to provide probabilistic location maps of the major molecular subtypes of gliomas, such as isocitrate dehydrogenase (IDH)-wild type versus IDH-mutant, IDH-mutant 1p19q co-deletion versus IDH-mutant non-1p19q co-deletion and IDH-wild-type O6-methylguanine-DNA methyltransferase (MGMT)-promoter methylation versus IDH-wild-type non-MGMT-promoter methylation. The relationship between glioma location and sex, age and overall survival is also examined.

## Materials and methods

### Data acquisition, storage and integration

Neuroimaging data of patients with glioma were accessed through several databases: UCSF-PDGM, UPENN-GBM, IvyGAP and TCGA were accessed through the Cancer Imaging Archive (TCIA; www.cancerimagingarchive.net), EGD was accessed through XNAT (https://xnat.health-ri.nl/data/archive/projects/egd), CPTAC-GBM and ACRIN were accessed through the 2021 Brain Tumor Segmentation (BraTS) Challenge (www.synapse.org), and REMBRANDT was accessed through Neuroimaging Informatics Tools and Resources Clearinghouse (NITRC; www.nitrc.org). Data acquisition and neuroimaging preprocessing were performed outside the current study and are described in more detail in prior publications: ACRIN,^12^ CPTAC-GBM,^12^ EGD,^13^ IvyGAP,^14^ REMBRANDT,^15^ TCGA,^16^ UCSF-PDGM^17^ and UPENN-GBM.^18^ Segmentation methodology differed across datasets. Some cohorts (e.g. UCSF-PDGM and IvyGAP) used manual expert delineation, while others (e.g. BraTS/ACRIN, CPTAC, EGD and TCGA/UPENN) employed semi-automated or algorithmic pipelines with expert quality control. In addition, some datasets provided subcompartmental masks (enhancing tumour, necrosis and oedema), whereas others provided a single whole-tumour segmentation. For consistency, all available compartments were combined into a unified tumour mask for pooled analyses. All datasets comprised patients with clinically presenting gliomas. Incidentally detected gliomas were not included, as patients were enrolled in these cohorts at the time of clinical presentation or diagnosis. Information regarding presenting symptoms was not uniformly available across databases and therefore could not be systematically analysed. Data were included if (i) it was an original patient, (ii) there was an MRI T1 image and (iii) there was a segmentation of the tumour. Data were excluded if (i) the MRI image was a recurrent tumour (*n* = 78) and (b) the segmentation quality was deemed poor by two independent raters (A.Y. and J.G.).

### Tumour segmentation

All databases provide tumour segmentation, and as such, individual manual tumour segmentation was not required. For all subsequent analyses, the provided brain tumour segmentation was used. Where the databases provide a more detailed division of tumour compartments, these subdivisions were combined into a single tumour segmentation to be compared across all patients (necrosis plus tumour).

### MRI image processing

All structural MRI images were preprocessed using minc-bpipe (https://github.com/CoBrALab/minc-bpipe-library), which performed non-uniformity correction and brain extraction. While masking the tumour using the provided segmentation, the individual extracted brains were non-linearly registered to Montreal Neurological Institute (MNI) space (MNI ICBM 2009b NLIN asymmetric)^19^ using Advanced Normalization Tools.^20^ All processing and registration steps were visually inspected in detail and quality controlled for each individual brain (A.Y. and J.G.).

### Generation of probabilistic maps and spatial analyses

All statistical analyses were performed using R (v4.0.2; https://www.r-project.org) and RMINC (https://github.com/Mouse-Imaging-Centre/RMINC). Voxel-wise regression analyses were performed to investigate the relationship between location and subject (e.g. age) and tumour characteristics (e.g. molecular marker). All tumours were divided into three equal groups (tertiles) based on their volumetric distribution within this cohort. Analyses with clinical attributes (age, sex and overall survival) were conducted using separate voxel-wise regression models for each attribute. Attributes were not included simultaneously in a multi-variable model, and no interaction terms or ANOVA analyses were applied. To characterize voxel-wise frequency of tumour occurrence in relation to these characteristic, sum-maps were created by adding up the individual tumour maps (tumour label = 1, background = 0). To test if specific brain regions are preferentially associated with tumour of different molecular subtypes, we used the Harvard-Oxford Atlas (http://www.cma.mgh.harvard.edu/) and performed a region-of-interest (ROI) analysis for the 138 ROIs included. Figures were created using Adobe Illustrator (v28.6; https://illustrator.adobe.com). Voxel-wise logistic regression was performed to compare tumour location across molecular and clinical subgroups. Multiple comparisons were addressed using false discovery rate (FDR) correction, and significance was defined at *P* < 0.001.

## Results

### Dataset and patient demographics

In total, 2164 patients were included (Supplementary Table 1): ACRIN (*n* = 3), CPTAC-GBM (*n* = 33), EGD (*n* = 774), IvyGAP (*n* = 29), REMBRANDT (*n* = 48), TCGA-GBM/LGG (*n* = 166), UCSF-PDGM (*n* = 500) and UPENN-GBM (*n* = 611). The main molecular subtype categories are as follows: IDH-wild type (*n* = 1324), IDH-mutant (*n* = 337), IDH-wild-type MGMT methylated (*n* = 403), IDH-wild-type MGMT non-methylated (*n* = 312), IDH-mutant 1p19q co-deleted (*n* = 102) and IDH-mutant non-1p19q co-deleted (*n* = 205). Demographic data are as follows (Supplementary Table 2): age at MRI (*n* = 1836; *x̄* = 58.83 ± 14.3), sex (*n* = 2123; female: 833, male: 1290) and overall survival (*n* = 944; 478.9 ± 485.1).

**Figure 1:**
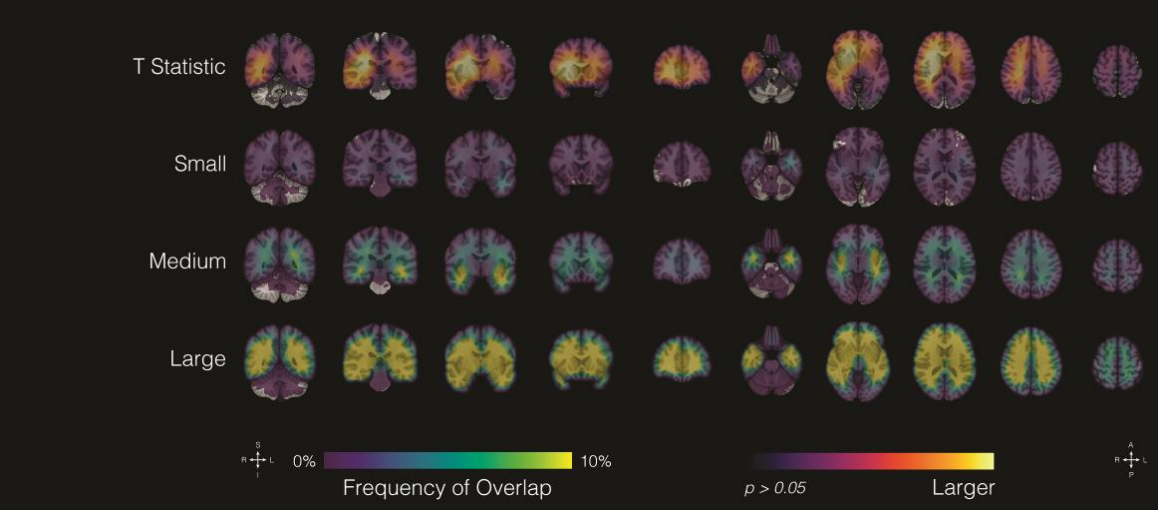
**Glioma volume and location.** Probabilistic maps showing the spatial distribution of gliomas stratified by tumour size. Tumours were divided into three equal groups: small (<25.2 ml, *n* = 721), medium (25.2–65.1 ml, *n* = 723) and large (>65.1 ml, *n* = 720). Results are displayed on coronal (left) and axial (right) sections of the T1-weighted MNI template brain. The top row shows voxel-wise *t*-statistics from regression of tumour volume on location (df = 2162), thresholded at uncorrected *P* < 0.001 (*t* ≥ 3.29) and *P* < 0.05 (*t* ≥ 1.96). The bottom three rows show frequency of tumour overlap in each size group, expressed as percentages. Scales indicate *t*-values (row 1) and overlap frequency (rows 2–4). Experimental unit: individual patients with glioma.

The initial analysis involved investigating the effect of glioma size on location (Fig. 1). To examine robustness, we generated distribution maps for the largest individual datasets (UCSF-PDGM, UPENN-GBM, TCGA and EGD; Supplementary Fig. 1). While dataset-specific effects are evident, overall spatial patterns were qualitatively consistent, supporting the validity of the pooled analyses. All tumours were divided into three equal groups based on their size: small (<25.2 ml), medium (25.2–65.1 ml) and large (>65.1 ml). In particular, small tumours had a predilection for left temporal regions, medium-sized tumours were found most frequently in the medial frontoparietal and bilateral temporal regions, and large tumours had a predilection for frontotemporoparietal regions, with a greater frequency of occurrence on the right relative to the left hemispheres.

**Figure 2:**
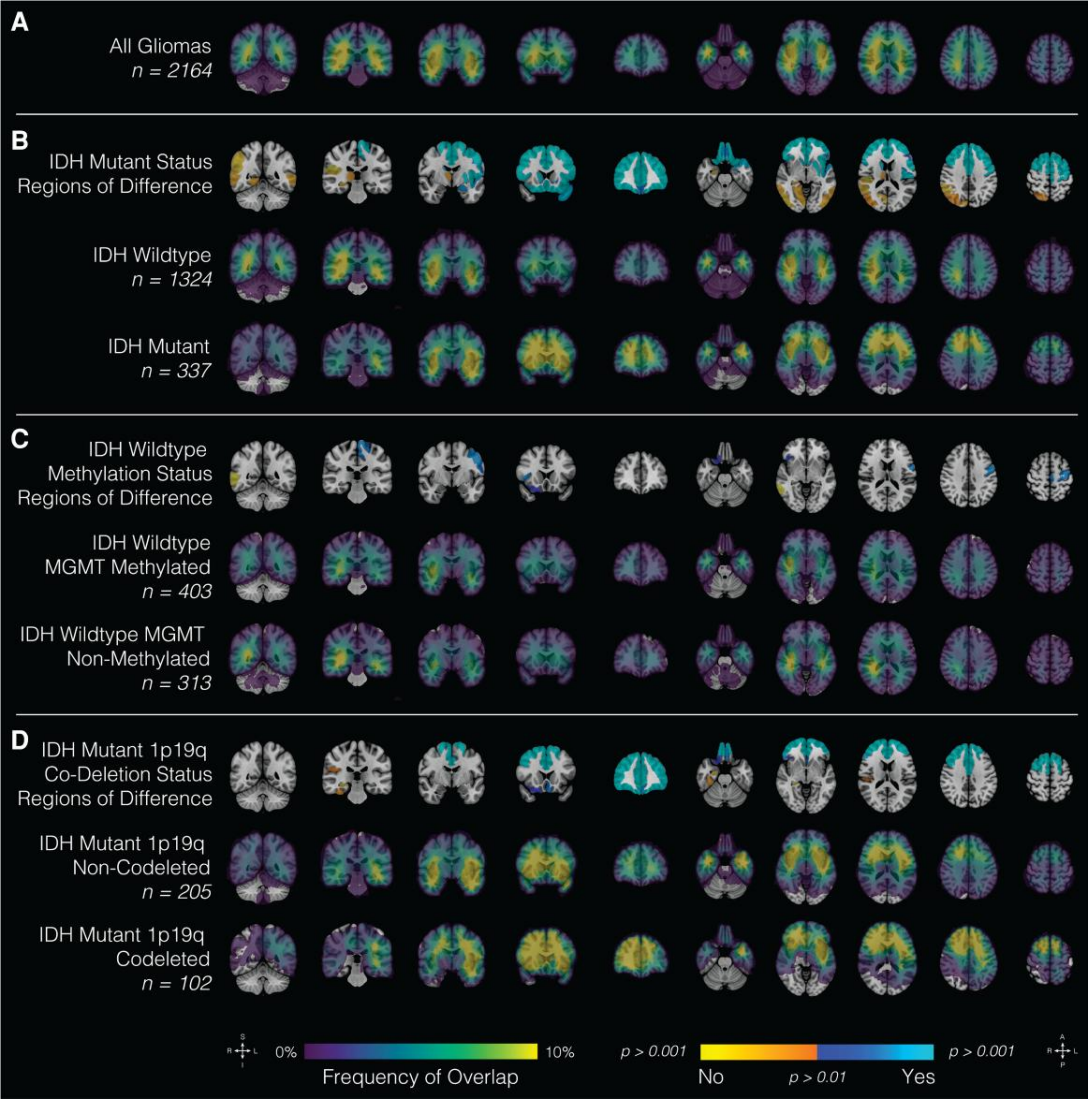
**Glioma type and location.** Probabilistic maps showing spatial distribution of all gliomas (**A**) and by molecular subtypes: (**B**) IDH-mutant status, (**C**) IDH-wild-type methylation status and (**D**) IDH-mutant 1p19q co-deletion status. Results are overlaid on the coronal (left half of the image) and axial (right half of the image) T1-weighted MNI brain. Scales indicate *t*-values (first row) and frequency of tumour overlap in percentages (remainder of the rows). IDH, isocitrate dehydrogenase; MNI, Montreal Neurological Institute; T1-weighted, T1-weighted magnetic resonance imaging; 1p19q, co-deletion of chromosome arms 1p and 19q.

The relationship between spatial landscape and well-characterized molecular markers was next examined (Fig. 2). Tumours that were IDH-wild type had a greater likelihood of growth in medial parietotemporal regions, while IDH-mutant tumours were more likely to be found in frontotemporal regions, with greater incidence of left-sided lesions. The regions of differential spatial patterns associated with IDH mutation status of the highest statistical significance (*P* < 0.001) were primarily cortical relative to subcortical regions, particularly left frontal (Fig. 2A). Discrete regions of differential spatial predilection by MGMT methylation status in IDH-mutant tumours were also noted and include left frontal and right temporal regions (Fig. 2B). Lastly, significant spatial differences were noted in 1p19q deleted relative to 1p19q non-co-deleted IDH-mutant tumours (Fig. 2C). Notably, 1p19q co-deleted tumours were more likely to be found in right frontotemporal and left temporal regions, although there was the general finding of bilaterally frontal infiltration of these tumours.

**Figure 3:**
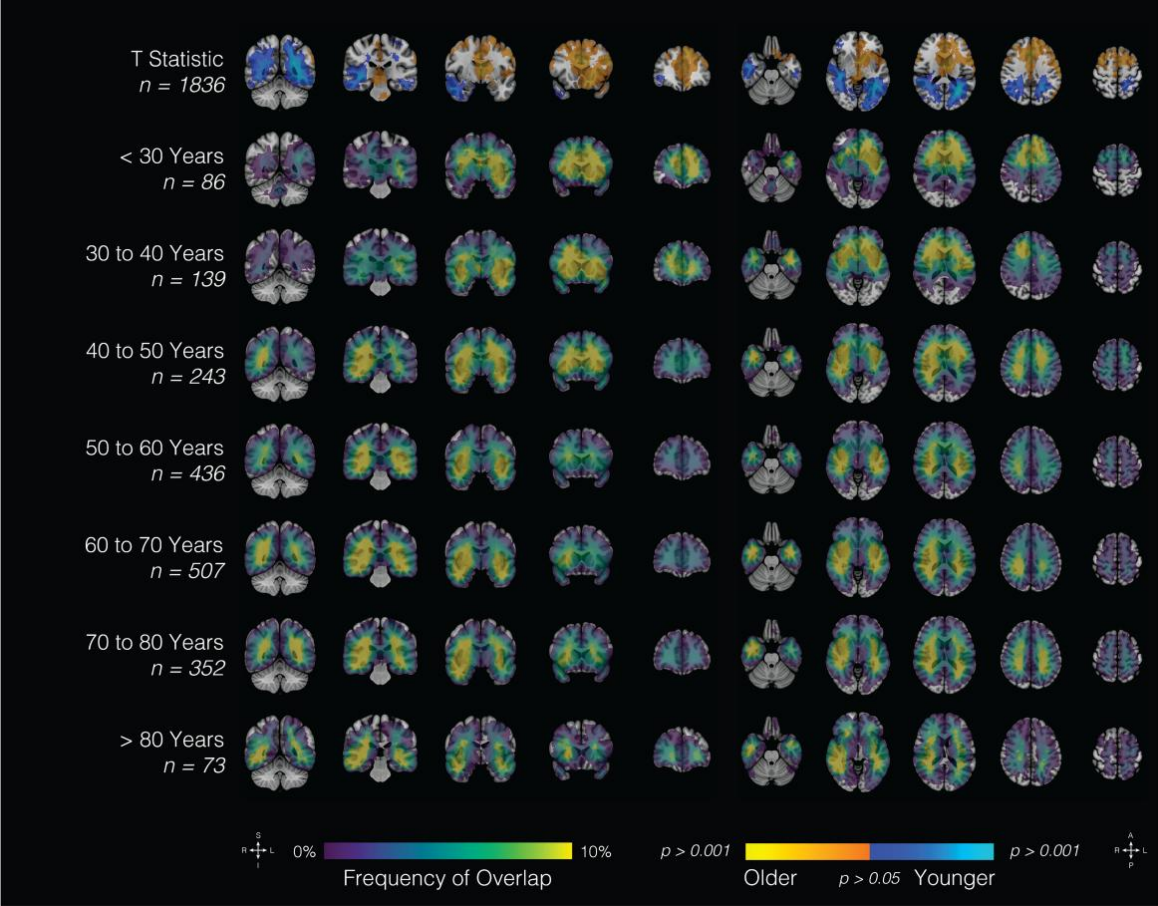
**Age at MRI and location.** Probabilistic maps showing spatial distribution of gliomas by age (*t*-statistic, continuous variable). Results are overlaid on the coronal (left half of the image) and axial (right half of the image) T1-weighted MNI brain. Scales indicate *t*-values (first row) and frequency of tumour overlap in percentages (bottom seven rows). MNI, Montreal Neurological Institute.

**Figure 4:**
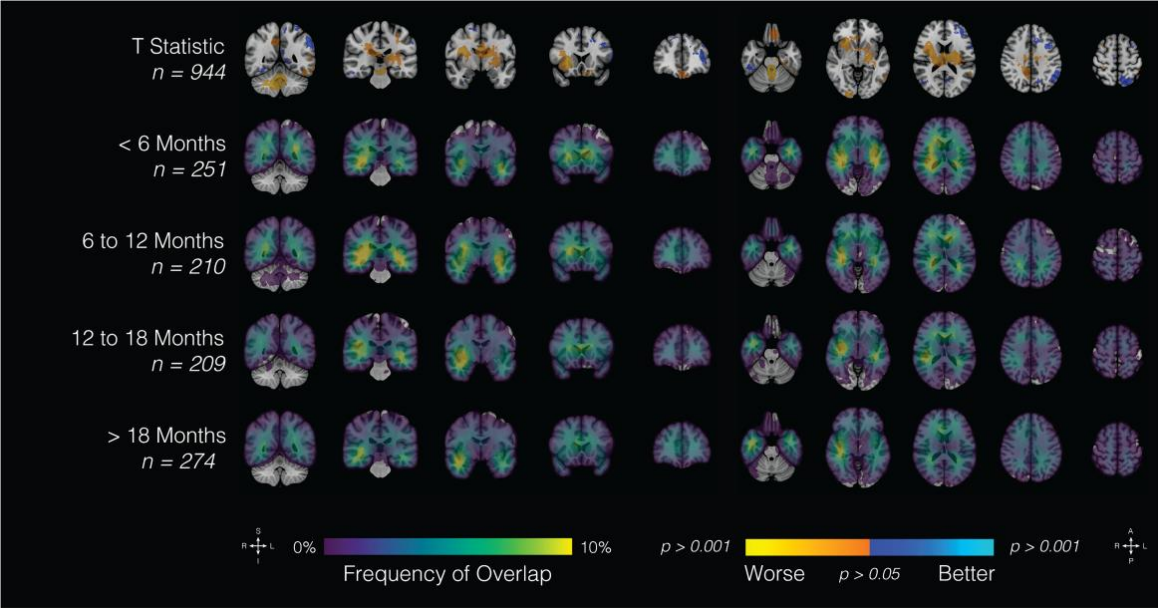
**Overall survival and location.** Probabilistic maps showing spatial distribution of gliomas by survival. Results are overlaid on the coronal (left half of the image) and axial (right half of the image) T1-weighted MNI brain. Scales indicate *t*-values (first row) and frequency of tumour overlap in percentages (bottom four rows). MNI, Montreal Neurological Institute.

**Figure 5:**
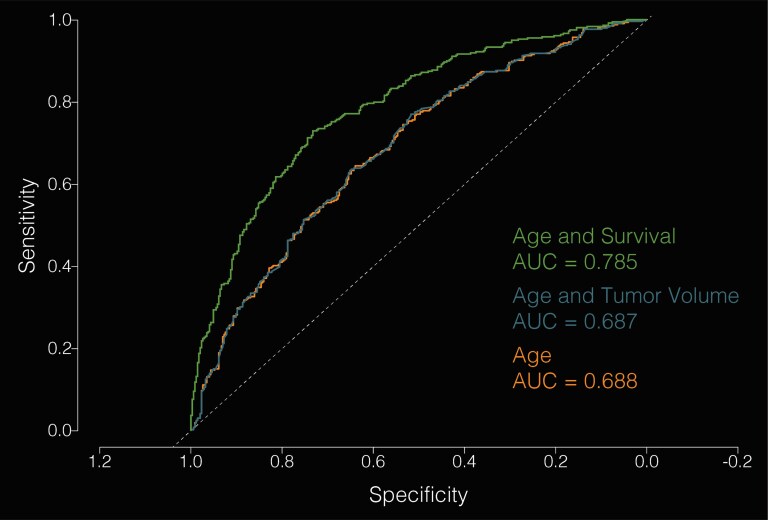
**A predictive model of survival based on anatomic tumour location and clinical characteristics.** Receiver operating characteristic (ROC) curves illustrating classification accuracy for predicting survival >1 year or <1 year (the group median) using three models: a model incorporating age and tumour overlap with the voxel-wise survival map [area under the curve (AUC) = 0.785], a model using age and tumour volume (AUC = 0.687) and a model using age alone (AUC = 0.688). Incorporating tumour location substantially improves prediction accuracy, whereas tumour size does not, suggesting that location may be a useful metric for survival prediction. Pairwise comparisons of ROC curves were performed using DeLong’s test: age + location versus age + volume (*Z* = 6.03; *P* = 1.66 × 10^−9^), age + location versus age alone (*Z* = 5.99; *P* = 2.16 × 10^−9^) and age + volume versus age alone (*Z* = −1.13; *P* = 0.257).

Clinical attributes were subsequently correlated with spatial maps. Patients were stratified among age categories by decade of life, as well as sex (Fig. 3; Supplementary Fig. 2). Younger patients tended to present with frontal tumours (R > L) as well as tumours involving medial structures; however, there were no significant sex-related differences in topographic location. Increasing age was associated with a higher parieto-occipital tumour burden. Patients with the lowest overall survival (<6 months) tended to present with tumours involving medial structures and parietal lobes bilaterally (Fig. 4). Patients with right temporal tumours, on average, tended to have higher rates (>18 months). In aggregate, tumour topography informed survival prediction relative to tumour volume (Fig. 5).

## Discussion

This study provides an up-to-date and comprehensive spatial map for gliomas and garners unique insights into the relationship between tumour location and clinical and molecular characteristics. With the implementation of the WHO 2021 brain tumour classification system, there has been a marked shift in characterizing gliomas, with a nearly sole emphasis on molecular markers, in contrast with the WHO 2016 categorization system, which mostly relied on histopathology. By mapping the spatial distribution of gliomas, the findings from this study demonstrate that gliomas exhibit a non-random spatial distribution within the brain. The findings from this study highlight several significant relationships between location and clinical and molecular features of glioma. Interestingly, tumour size was correlated with neuroanatomical regions. This may be due to several factors, including but not limited to a threshold in size that a tumour needs to reach before becoming symptomatic, involvement of eloquent brain areas or impact on neurophysiological function. Studies investigating tumour growth velocity may be helpful in further elucidating this. Studies have demonstrated that disruption in functional connectivity in the setting of high-grade gliomas may carry prognostic value and inform treatment planning.^20^ As such, it is anticipated that larger tumours may infiltrate and impact multiple functional networks, thereby negatively impacting patient survival. It is possible that functional connectivity, including white matter tracts, may differentially facilitate or limit distant spread.

The findings from this study also demonstrate distinct spatial patterns of glioma molecular subtypes. This is consistent with previous reports in smaller case series demonstrating distinct local distribution of glioma by molecular markers.^21,22^ Our results could be integrated into future radiology workflows serving as a prospective diagnostic aid. In particular, in cases where biopsy may be a limiting factor to tissue access, or in the emerging area of liquid biopsies, molecular–radiographic correlations have the potential to aid in the clinical decision-making.

The correlation between tumour molecular classification and survival is well characterized. To further understand the relationship between tumour location and other clinical features that may correlate with molecular markers, our results show that patients with right temporal tumours, on average, tend to have higher survival rates, possibly related to the predilection for certain types of molecular pathology. Our molecular analyses demonstrated that these tumours were most likely to be 1p19q co-deleted, which is consistent with the known improved prognosis of these tumours relative to other molecular glioma subtypes.^23^

Several limitations should be noted. The findings of clinical attributes and tumour laterality may be biased by patients who did not undergo surgery, such as patients with non-operative infiltrative tumours in eloquent regions and who were not candidates for awake craniotomy. Neuroimaging acquisition, including MRI systems, varied across databases. Furthermore, the MRI segmentation methodology also was heterogeneous. Acquisition of molecular and clinical data also differed across datasets. Finally, although dataset-specific effects are apparent (Supplementary Fig. 1), the use of a large, aggregated cohort aided in mitigating these potential sources of heterogeneity as such a limitation would be particularly relevant for smaller studies restricted to single datasets. In aggregate, this underscores the advantage of a pooled multi-dataset approach. Further investigations could expand upon the current study to include a broader range of datasets and methodologies to provide a more comprehensive understanding.

## Conclusion

Probabilistic spatial mapping of gliomas is a powerful tool to gain mechanistic insights into tumour pathogenesis, improve radiological interpretation, predict clinical outcomes and develop personalized treatment approaches for these tumours. The unique microenvironmental features of different brain regions likely influence glioma initiation and progression. This large-scale study leverages a comprehensive dataset to generate high-fidelity probabilistic maps of tumour locations, provides a radiographic resource for future research and highlights the unique spatial patterns of glioma based on clinical and molecular features.

## Supplementary Material

fcaf459_Supplementary_Data

## Data Availability

All data generated or analysed during this study are linked in this article in the Material and Methods section. Further inquiries can be directed to the corresponding author.
