# Supplementary material for: Data-driven probabilistic mapping of the spatial and molecular landscape of glioma
Source: Brain Commun. 2025 Nov 26;8(1):fcaf459. doi: 10.1093/braincomms/fcaf459 (PMC12914576; doi:10.1093/braincomms/fcaf459)
Supplement: fcaf459_Supplementary_Data [file fcaf459_supplementary_data.pdf]

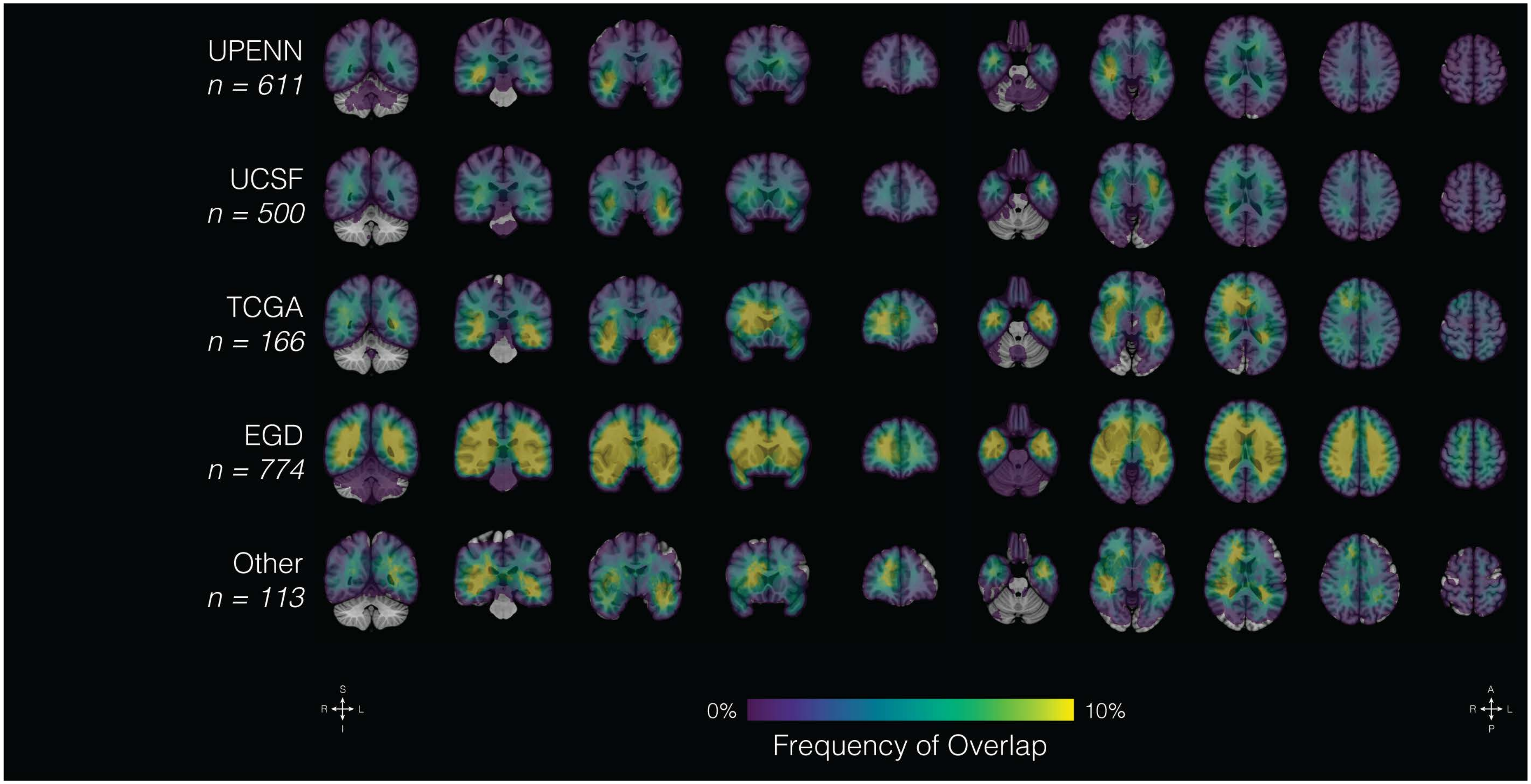

Supplementary Figure 1. Probabilistic maps showing spatial distribution of gliomas by dataset (t-statistic, continuous variable). Distribution maps for the three largest individual datasets (UCSF-PDGM, UPENN-GBM, and EGD). Results are overlaid on coronal (left half of the image) and axial (right half of the image) T1-weighted MNI brain. Colors indicate frequency of tumor overlap in percentages.

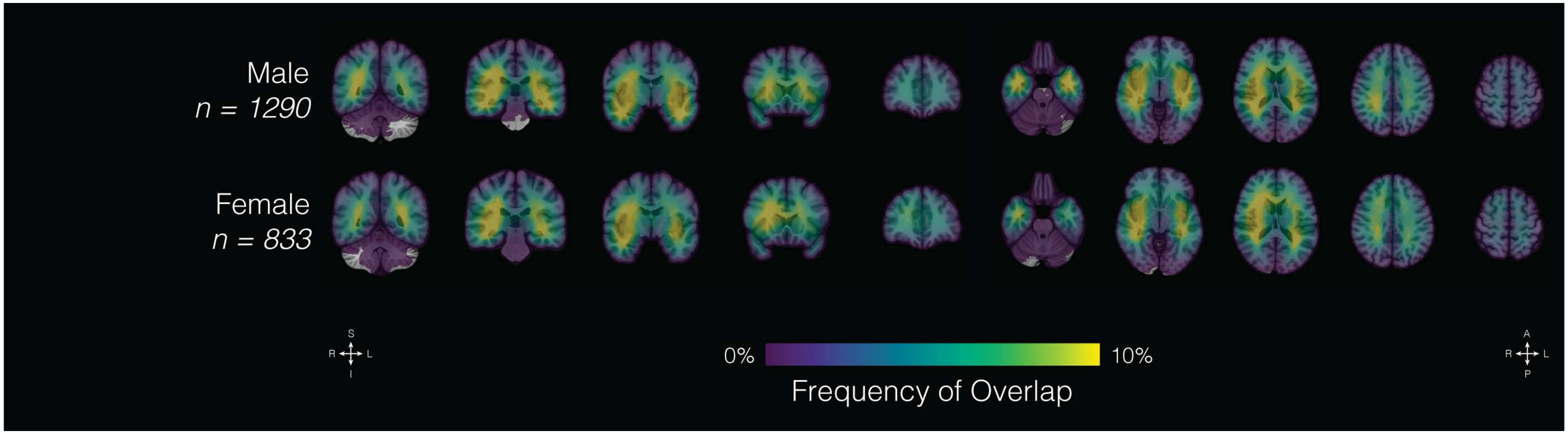

Supplementary Figure 2. Probabilistic maps showing distribution of gliomas by sex (t-statistic, continuous variable). Distribution maps by sex. Results are overlaid on coronal (left half of the image) and axial (right half of the image) T1-weighted MNI brain. Colors indicate frequency of tumor overlap in percentages.

Database	All Gliomas	IDH-Wildtype	IDH-Mutant	IDH-Wildtype MGMT Methylated	IDH-Wildtype MGMT Non-Methylated	IDH-Mutant 1p19q-Codeleted	IDH-Mutant Non-1p19q-Codeleted
**ACRIN**	3	N/A	N/A	N/A	N/A	N/A	N/A
**CPTAC-GBM**	33	N/A	3	N/A	N/A	N/A	N/A
**EGD**	774	312	155	N/A	N/A	73	77
**IvyGAP**	29	25	3	8	12	1	1
**REMBRANDT**	48	N/A	N/A	N/A	N/A	N/A	N/A
**TCGA**	166	91	57	26	40	13	43
**UCSF-PDGM**	500	397	103	265	109	15	84
**UPENN-GBM**	611	499	16	104	151	N/A	N/A
	∑ ***2164***	∑ ***1324***	∑ ***337***	∑ ***403***	∑***312***	∑ ***102***	∑ ***205***

Supplementary Table 1. Molecular subtypes of the datasets. ACRIN: American College of Radiology Imaging Network; CPTAC-GBM: Clinical Proteomic Tumor Analysis Consortium - Glioblastoma Multiforme; EGD: European Glioma Database; IDH: Isocitrate Dehydrogenase; IvyGAP: Ivy Glioblastoma Atlas Project; REMBRANDT: REpository for Molecular BRAin Neoplasia DaTa; TCGA: The Cancer Genome Atlas; UCSF-PDGM: University of California, San Francisco - Preoperative Diffuse Glioma; UPENN: University of Pennsylvania Glioblastoma; and 1p19q: Co-deletion of chromosome arms 1p and 19q.

**Database**	**Age (Years)**		**Overall Survival (Days)**		***Sex***	
	n	$\bar{x} \pm SD$	***n***	$\bar{x} \pm SD$	***n***	***F/M***
**ACRIN**	N/A	N/A	N/A	N/A	3	F0/M3
**CPTAC-GBM**	N/A	N/A	14	344.1 ± 256.6	19	F7/M12
**EGD**	725	56.7 ± 14.6	N/A	N/A	773	F281/M92
**IvyGAP**	N/A	N/A	20	488.7 ± 370.2	29	F15/M14
**REMBRANDT**	N/A	N/A	43	869.4 ± 1024.0	23	F10/M13
**TCGA**	N/A	N/A	165	577.1 ± 674.6	165	F75/M90
**UCSF-PDGM**	500	56.8 ± 15.0	250	456.1 ± 363.2	500	F201/M299
**UPENN**	611	63.0 ± 12.4	452	422.2 ± 355.3	611	F244/M367
	∑ ***1836***	***58.83 ± 14.3***	∑ ***944***	***478.9 ± 485.1***	∑ ***2123***	∑ ***F833/M1290***

Supplementary Table 2 Demographics of dataset. ACRIN: American College of Radiology Imaging Network; CPTAC-GBM: Clinical Proteomic Tumor Analysis Consortium - Glioblastoma Multiforme; EGD: European Glioma Database; F: female; IvyGAP: Ivy Glioblastoma Atlas Project; REMBRANDT: REpository for Molecular BRAin Neoplasia DaTa; M: male; TCGA: The Cancer Genome Atlas; UCSF-PDGM: University of California, San Francisco - Preoperative Diffuse Glioma; and UPENN: University of Pennsylvania Glioblastoma;
